# The Medical, Surgical, and Hygienic Exhibition

**Published:** 1902-05-31

**Authors:** 


### THE MEDICAL, SURGICAL, AND HYGIENIC EXHIBITION.

THIS annual exhibition was held at the Queen's Hall from May 20th to the 23rd inclusive, and attracted an amount of interest which justifies the verdict that it was the most successful exhibition that has yet been given by this Association. There was an appreciable increase in the attendance of medical men, who appeared to take advantage of the opportunity thus afforded them of familiarising themselves with the many new preparations and appliances that were on view.

The preparations of W. R. Warner and Co. were exhibited to advantage by Messrs. F. Newbery and Sons, and included chocolate digestive tablets, tono-sumbul cordial, and sugar-coated pills, while Mr. W. Martindale gave an interesting and varied display of drugs, sterilised dressings, etc. Messrs. Arthur and Co. were in evidence with bromaurum for rheumatism, etc., and Edgars, Limited, exhibited their proprietary lotion. New exhibits which attracted much attention were Peptenzyme, a digestant prepared in powder, tablets, and other forms; and Billons Ovo Lecithin, a di-stearo-glycero phosphate of choline, made from the yolk of egg. Messrs. Nicolay and Co. displayed Hommel's Hæmatogen, a well-known tonic, and the British Iron Milk Syndicate introduced Dolle's aromatic iron milk, a new hæmogenetic. Maltova, a valuable preparation, composed of extract of malt and fresh eggs was much in evidence, and deserves special mention. Attention should be called to Virol, the well-known infant and invalid food, which has been favourably received at hospitals, and a large supply of which accompanies the present Antartic Expedition. Bovril was also exhibited in various convenient forms.

Diabetic foods were exhibited by Messrs. Callard and Co., preserved fruits and marmalade being prominent, Cheltine Foods, Limited, showed Cheltine powdered milk, and the Manhu Food Company, Limited, diabetic flour. Plasmon food preparations claimed particular attention, especially the diabetic biscuits, which are a recent production. The Hovis Company gave an exhibit of their foods, while the Protene Company secured considerable notice with their milk proteid manufactures, which obtained the only gold medal awarded for milk proteids at the last Paris Exhibition. Important exhibits of milk preparations were shown by the Aylesbury Dairy Company, and Messrs. Welford and Co., and included their humanised and sterilised milks, so well known to the medical profession. Condensed milk, and milk foods were shown by Mr. Henri Nestlé and the Angle-Swiss Milk Company, and attracted considerable attention. Marvis, a prepared fish food, was shown in several forms, and the Myosin Albumen Meat Extract Company exhibited "Mamesin," a beef preparation.

Messrs. Cosenza and Co.'s stall contained Maggi's Consommé, Bouillon, Soups, and Gluten Cocoa, which have attained much popularity. Liebig's Extract of Meat Company gave an exhibit of Lemco and Oxo, while Hipi, an excellent mutton essence, was shown by Messrs. Nelson, Dale and Co.

Turning to mineral waters, "Rosbach," a high-class table water of much delicacy, attracted marked attention. Messrs. Ingram and Royle exhibited a number of medicinal waters, including Vichy, Carlsbad, and the well-known Hunyadi János. Messrs. Cooper and Co., of Gloucester Road, S.W., had an extensive show of their Globenaris medicinal and table waters, which are supplied in porcelain-lined syphons and are of a high class.

Messrs. Greenlees Brothers gave an exhibit of their Scotch and Irish Whiskies of superior quality, and Messrs Marshall and Elvy of "Satinette" and Courvosier's Brandy.

Surgical instruments and appliances were extensively shown by Messrs. Maw, Son and Sons, The Holborn Surgical Instrument Company, and by Mr. W. K. Stacy, who also had a fine display of nurses' Wallets and other requisites. Much attention was devoted to the "Gunthorp" appliances for deformed feet of the O'Connor Extension Company, and to the Instep Arch Sock for flat foot, shown by Mr. Thomas Holland. The prominent exhibit of Hartmann's Antiseptic Wood Wool manufactures attracted medical men and nurses to a marked degree, and the Cellular Clothing Company's stall, where a variety of Aertex Cellular Clothing was displayed, afforded considerable interest.

The electrical exhibits formed a prominent feature of the exhibition. The General Electric Company had an interesting exhibit including X-ray and electro-medical apparatus, as also had Messrs. H. W. Cox, Limited. The Sanitas Electrical Company attracted general notice with their electric bath and the "Dermo" lamp for the production of ultra-violet rays. The Dowsing Radiant Heat Company gave an extensive exhibit of baths and apparatus for the application of the now universally adopted radiant heat and light treatment. The Muschik Vibratorium apparatus for treatment by vibration was in operation.

The Berkefeld Filter Company showed their well-known filters for hospital and household use. Messrs. J. Defries and Sons exhibited their Pasteur filter, and called special attention to the "Equifex" Odourless Tank Wagon, an ingenious apparatus for rapidly emptying cesspools, etc., by which offence or risk of infection is reduced to a minimum.

The Gem Supplies Company exhibited a bath cabinet with a well-protected three-light stove, and the Gem Pure Water Still, a domestic apparatus for distilling water.

Publications were shown by Mr. Henry Kimpton, Messrs. Rebman, Limited (whose beautifully executed illustrations and diagrams attracted general notice), and by the Scientific Press, Limited, who exhibited a number of medical works and Standard Text-books on Nursing. The well-known temperature charts of Messrs. Wodderspoon and Co. were also exhibited.

With regard to disinfectants, soaps, etc., the Izal fluid, powder, oil, and toilet preparations of Messrs. Newton Chambers and Co., Limited, attracted much notice, while the Izal Perles for the internal employment of this disinfectant afforded special medical interest. Jeyes' fluid, creolin and powder were well in evidence, Dr. Rideal's Anti-typhoid Tablets (for internal use) being included in this exhibit. Oowana, Limited, attracted much notice with an excellent toilet soap of that name, the purity of which should commend it. Eucryl, Limited, showed Bath Eucryl (a refreshing addition to the bath), and toilet soaps and creams of a high quality.

Sanitary specialities and appliances for hospital use were exhibited in considerable variety and excellence by Messrs.

Doulton and Co., Limited, whose lavatory, with pedal action for water and waste, claims mention; and by Messrs. George Jennings, Limited, who also exhibited an ingenious air-tight apparatus for emptying and disinfecting bed-pans.

Messrs. Maple and Co. gave an interesting exhibit of hospital furniture, prominent in which was a ward table combined with shelves for medicines, and a lock-up poison cupboard. This exhibit included a number of "Di-ag-nl" bedsteads and cots by Messrs. John and Joseph Taunton, Limited, for hospital and asylum use. These bedsteads, which are well constructed and conveniently designed, have given satisfaction at a large number of institutions.

